# Supplementary material for: RNA-Seq Gene Profiling - A Systematic Empirical Comparison
Source: PLoS One. 2014 Sep 30;9(9):e107026. doi: 10.1371/journal.pone.0107026 (PMC4182317; doi:10.1371/journal.pone.0107026)
Supplement: File S1 — Supplementary tables and figures. (PDF) [file pone.0107026.s001.pdf]

RNA-seq gene profiling - a systematic comparison

Supplementary material

Nuno A. Fonseca John C. Marioni

Alvis Brazma

European Molecular Biology Laboratory

European Bioinformatics Institute (EMBL-EBI)

Wellcome Trust Genome Campus

Hinxton, Cambridge CB10 1SD

United Kingdom

August 29, 2014

List of Tables

S1 Main parameters 4
S2 Mappers: support for splicing 4
S3 Experimental data sets. 4
S4 Synthetic data sets 5
S5 Average rankings of the pipelines across the data sets with single-end reads. 12
S6 Average rankings of the pipelines across the data sets with paired-end reads 13
S7 List of genes with consistent high error. 18
S8 GO terms of the genes with consistent high error (1/2). 19
S9 GO terms of the genes with consistent high error (2/2). 20

List of Figures

S1 Experimental RNA-seq data from Human - SRP000225 6
S2 Experimental RNA-seq data from mouse - E-MTAB-599 . . . 7
S3 Experimental RNA-seq data from E. coli K12 - E-MTAB-387. 8
S4 Distribution of the error across all data sets and pipelines segmented by pipelines using spliced and unspliced aligners. . . . 9
S5 Number of genes with high error (> 100%) or low error (< 10%) 10
S6 Number of results gathered by pipeline, read length and library tag type (SE=single end, PE=Paired-end). 11

S7 Error by number of reads (normalized per gene using the sum of the transcript lengths of the gene and sequencing depth of the data set). 14
S8 Percentage of the gene length "explained" by exons with a length shorter than 200 nucleotides. 15
S9 Gene sequence uniqueness: $\frac{1}{N}$, where N is the number of locations in the genome similar to the gene's sequence. 16
S10 Number of genes with a positive or negative error across all data sets and pipelines. 17

Tool	Version	Main Parameters
BWA1	0.6.2	
BWA2	0.6.2	
Bowtie1	0.12.9	–fullref –sam -q –best –strata -k 10
Bowtie2	2.0.5	–end-to-end -k 10
Cufflinks1	1.3.0	–min-isoform-fraction 0.05 –multi-read-correct -G
Cufflinks2	2.0.0	–min-isoform-fraction 0.05 –multi-read-correct -G
Flux-Capacitor	1.2.3-20121215021902	
GSNAP	2012-07-20	-N 1 -A sam
HTSeq	0.5.3p9	htseq-count -i gene_id –mode=(union—intersection-nonempty) –stranded=no
OSA	2.0.1	-alignrna SearchNovelExonJunction=True
Smalt	0.6.4	-f samsoft
Star	2.2.0	-outFilterMultimapNmax 10 –sjdbOverhang 20 –sjdbFileChrStartEnd
TopHat1	1.4.1	–min-intron-length 6
TopHat2	2.0.6	–no-coverage-search –min-intron-length 6

Table S1: Aligners and quantification methods: versions and parameters used.

Mapper	Splicing
BWA1	No
BWA2	No
Bowtie1	No
Bowtie2	No
GSNAP	Yes
OSA	Yes
Smalt	No
Star	Yes
TopHat 1	Yes
TopHat 2	Yes

Table S2: Mappers: support for splicing

Dataset	Species	Data	FASTQ	SE	PE	RL
E-MTAB-513	Human	16 organism parts	32	16	16	75 & 50
SRP000225	Human	2 organism parts	6	6	0	36
E-MTAB-599	Mouse	organism part (6)	36	36	0	76
E-MTAB-387	E.coli K12	2 developmental stages	2	2	0	36

Table S3: Experimental data sets.

Dataset	SE/PE	RL	Depth
l50.d10.se	SE	50	10
l100.d10.se	SE	100	10
l150.d10.se	SE	150	10
l200.d10.se	SE	200	10
l50.d10.pe	PE	50	10
l100.d10.pe	PE	100	10
l150.d10.pe	PE	150	10
l200.d10.pe	PE	200	10
l50.d30.se	SE	50	30
l100.d30.se	SE	100	30
l150.d30.se	SE	150	30
l200.d30.se	SE	200	30
l50.d30.pe	PE	50	30
l100.d30.pe	PE	100	30
l150.d30.pe	PE	150	30
l200.d30.pe	PE	200	30
l50.d60.se	SE	50	60
l100.d60.se	SE	100	60
l150.d60.se	SE	150	60
l200.d60.se	SE	200	60
l50.d60.pe	PE	50	60
l100.d60.pe	PE	100	60
l150.d60.pe	PE	150	60
l200.d60.pe	PE	200	60
l50.d120.se	SE	50	120
l100.d120.se	SE	100	120
l150.d120.se	SE	150	120
l200.d120.se	SE	200	120
l50.d120.pe	PE	50	120
l100.d120.pe	PE	100	120
l150.d120.pe	PE	150	120
l200.d120.pe	PE	200	120

Table S4: Synthetic data sets. Each simulated data set is composed by 8 fastq files for which the true number of raw counts per gene is known. The SE/PE column indicates if the pairing of the reads (SE-single end, PE- paired-end), the RL column indicates the read length and Depth the sequencing depth.

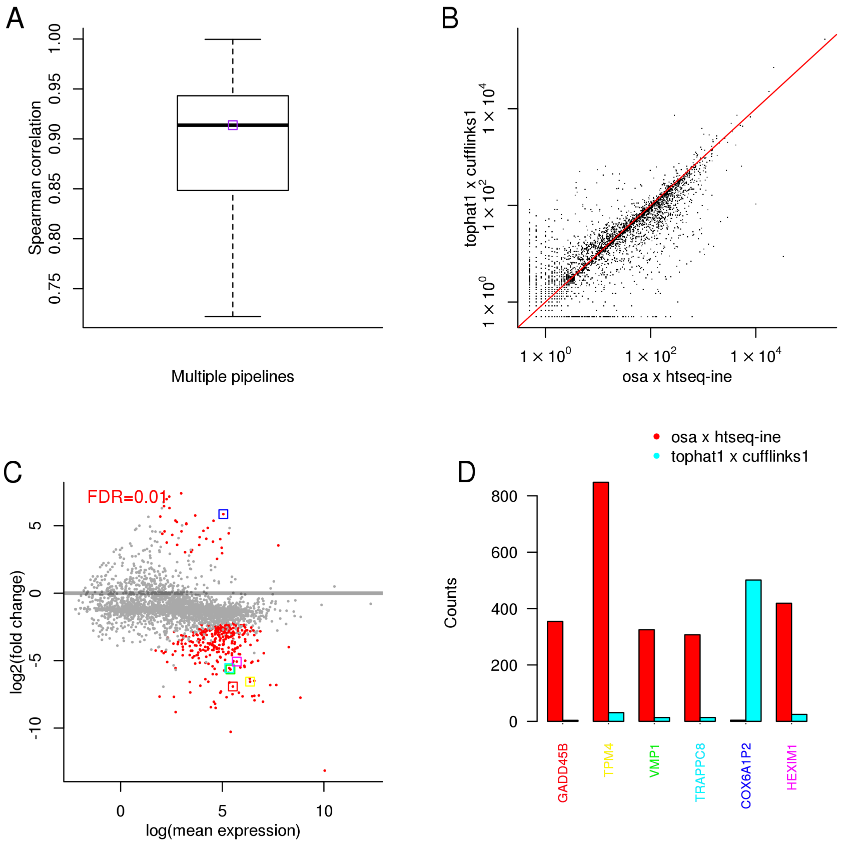

Figure S1: Experimental RNA-seq data from Human - SRP000225. A) Spearman correlation distribution between the gene expression profiles inferred by different pipelines; B) correlation between two specific pipelines (the respective Spearman correlation is shown in plot A as a purple box); C) fold change between the gene expression values inferred by the same two pipelines - dots in red denote genes where the expression values are significantly different between the two selected pipelines (for a false discovery rate of 0.01); D) expression values inferred by the two pipelines for the six selected (boxed) genes in plot C).

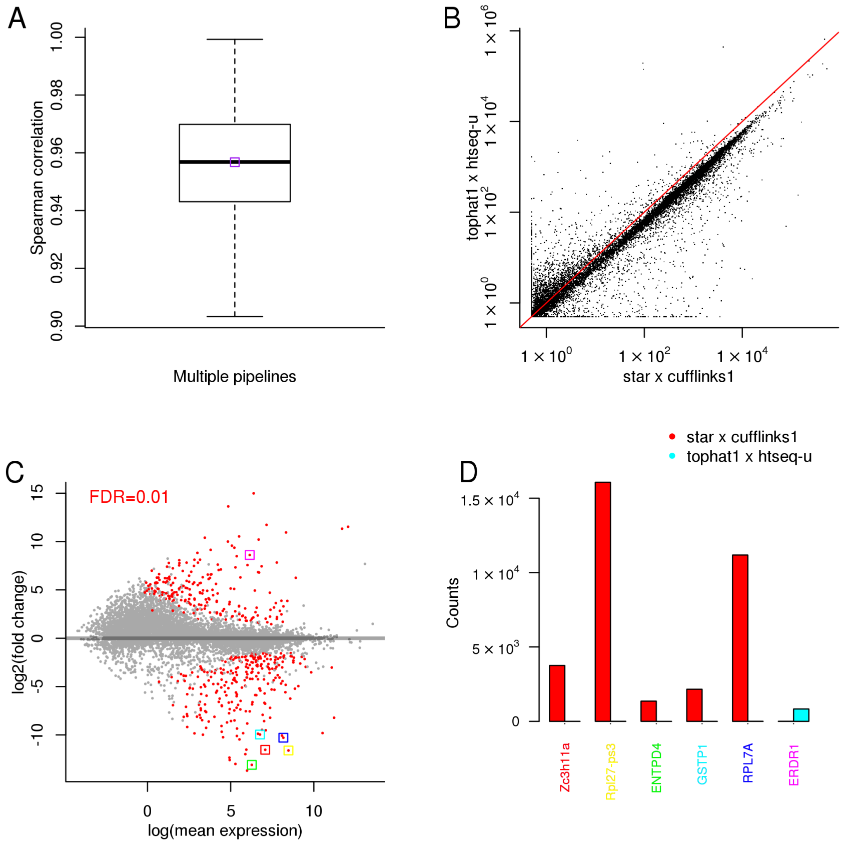

Figure S2: Experimental RNA-seq data from mouse - E-MTAB-599. A) Spearman correlation distribution between the gene expression profiles inferred by different pipelines; B) correlation between two specific pipelines (the respective Spearman correlation is shown in plot A as a purple box); C) fold change between the gene expression values inferred by the same two pipelines - dots in red denote genes where the expression values are significantly different between the two selected pipelines (for a false discovery rate of 0.01); D) expression values inferred by the two pipelines for the six selected (boxed) genes in plot C).

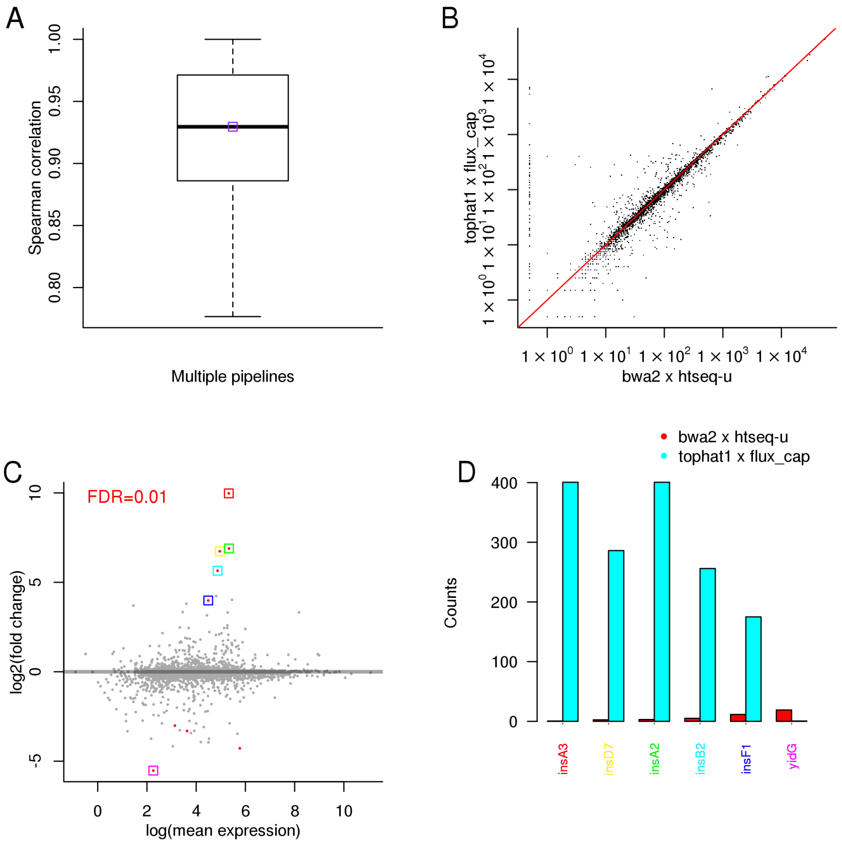

Figure S3: Experimental RNA-seq data from E. coli K12 - E-MTAB-387. A) Spearman correlation distribution between the gene expression profiles inferred by different pipelines; B) correlation between two specific pipelines (the respective Spearman correlation is shown in plot A as a purple box); C) fold change between the gene expression values inferred by the same two pipelines - dots in red denote genes where the expression values are significantly different between the two selected pipelines (for a false discovery rate of 0.01); D) expression values inferred by the two pipelines for the six selected (boxed) genes in plot C).

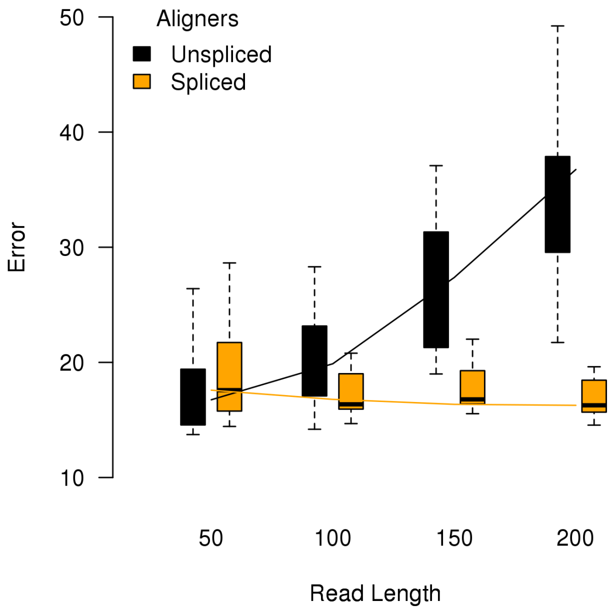

Figure S4: Distribution of the error across all data sets and pipelines segmented by pipelines using spliced and unspliced aligners.

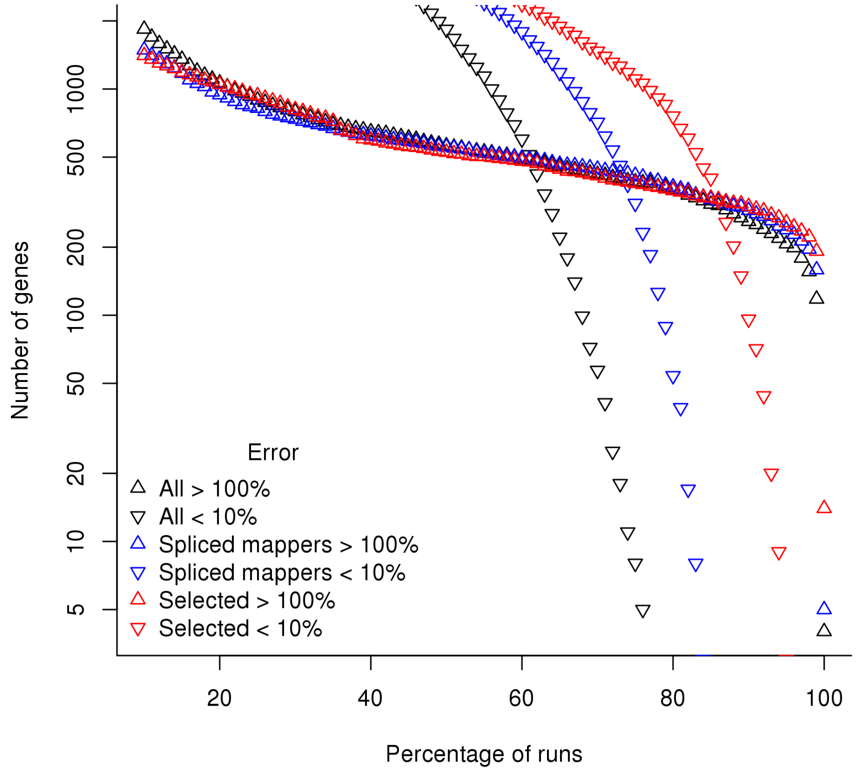

Figure S5: Number of genes with high error (> 100%) or low error (< 10%) across all data sets and: i) all pipelines; ii) pipelines with spliced aligners; iii) pipelines combining OSA or Tophat1 with htseq-ine, Cufflinks2, and Flux-capacitor.

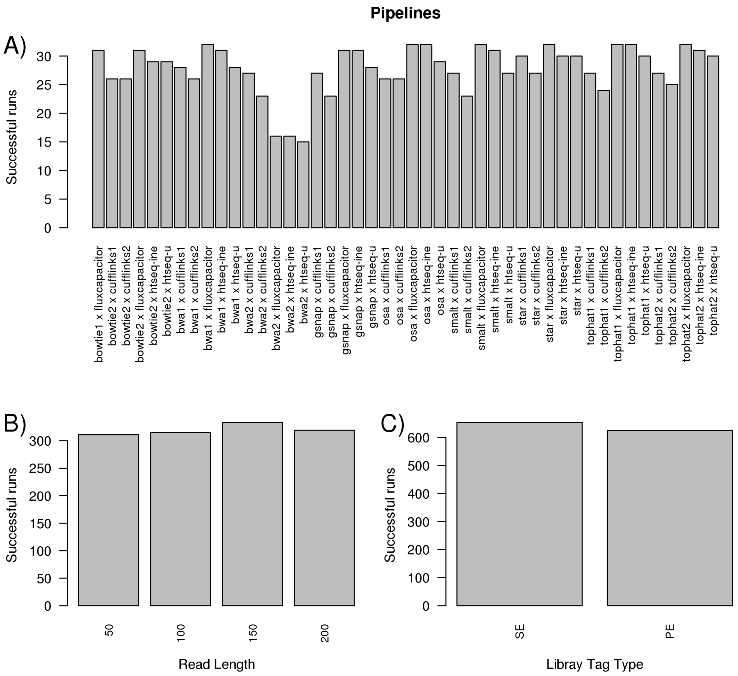

Figure S6: Number of results gathered by pipeline, read length and library tag type (SE=single end, PE=Paired-end).

Pipeline		Overall	Error		Spearman	
Aligner	Quant. Method	Rank	Rank	mean ± sd	Rank	mean ± sd
osa	htseq-ine	20	9	16.09 ± 0.68	11	0.93 ± 0.01
tophat1	htseq-ine	23	11	16.92 ± 2.58	13	0.93 ± 0.01
smalt	htseq-ine	24	15	18.35 ± 8.24	9	0.94 ± 0.00
osa	fluxcapacitor	26	24	19.38 ± 0.87	2	0.95 ± 0.00
tophat2	htseq-ine	26	14	18.91 ± 6.8	12	0.93 ± 0.01
star	fluxcapacitor	27	23	19.12 ± 0.9	4	0.94 ± 0.00
star	htseq-ine	27	10	16.84 ± 2.8	16	0.93 ± 0.01
bwa2	htseq-ine	28	16	20.34 ± 6.05	12	0.93 ± 0.02
gsnap	htseq-ine	31	15	22 ± 10.23	17	0.93 ± 0.01
tophat1	fluxcapacitor	31	25	19.54 ± 0.92	5	0.94 ± 0.00
tophat2	fluxcapacitor	33	27	19.98 ± 1.28	6	0.94 ± 0.01
smalt	htseq-u	35	21	20.98 ± 9.93	14	0.93 ± 0.00
star	cufflinks2	35	8	15.65 ± 0.83	27	0.91 ± 0.01
tophat1	cufflinks2	35	9	22.03 ± 20.29	25	0.91 ± 0.04
bwa2	fluxcapacitor	36	26	20.84 ± 2.99	10	0.94 ± 0.01
osa	htseq-u	36	16	22.84 ± 19.12	21	0.92 ± 0.03
bwa2	htseq-u	37	20	23.75 ± 13.71	17	0.92 ± 0.02
gsnap	fluxcapacitor	37	28	21.92 ± 8.47	10	0.94 ± 0.01
gsnap	htseq-u	38	15	21.18 ± 10.27	23	0.92 ± 0.01
tophat1	htseq-u	38	18	18.77 ± 4.73	20	0.92 ± 0.00
star	htseq-u	39	15	16.99 ± 2.58	23	0.92 ± 0.00
tophat1	cufflinks1	39	11	16.09 ± 0.83	29	0.91 ± 0.01
tophat2	htseq-u	39	19	20.66 ± 8.61	20	0.92 ± 0.01
bwa2	cufflinks2	41	21	21.92 ± 7.76	20	0.92 ± 0.02
osa	cufflinks2	41	14	20.51 ± 9.56	27	0.91 ± 0.03
bwa1	htseq-ine	44	23	24.58 ± 8.97	21	0.91 ± 0.03
osa	cufflinks1	44	15	18.31 ± 6.03	30	0.91 ± 0.01
star	cufflinks1	44	13	17.35 ± 4.49	31	0.91 ± 0.01
tophat2	cufflinks2	44	16	27.4 ± 25.32	29	0.88 ± 0.11
gsnap	cufflinks2	45	17	23.55 ± 16.98	27	0.9 ± 0.05
smalt	fluxcapacitor	46	28	22.97 ± 7.7	18	0.93 ± 0.01
bwa1	htseq-u	49	25	25.64 ± 9.59	24	0.9 ± 0.03
bwa1	fluxcapacitor	50	31	26.57 ± 8.65	20	0.91 ± 0.04
gsnap	cufflinks1	52	20	24.56 ± 15.57	32	0.91 ± 0.01
smalt	cufflinks2	52	27	28.88 ± 21.21	25	0.9 ± 0.06
tophat2	cufflinks1	52	20	27.16 ± 19.91	32	0.91 ± 0.01
bwa1	cufflinks2	54	26	33.52 ± 23.29	28	0.87 ± 0.09
smalt	cufflinks1	54	28	29.34 ± 21.16	26	0.9 ± 0.08
bwa2	cufflinks1	58	28	33.82 ± 22.89	30	0.86 ± 0.08
bwa1	cufflinks1	59	28	38.15 ± 29.03	31	0.85 ± 0.11
bowtie2	htseq-ine	61	26	23.11 ± 9.45	35	0.88 ± 0.01
bowtie1	fluxcapacitor	64	34	28.07 ± 7.37	30	0.89 ± 0.04
bowtie2	htseq-u	66	30	25.96 ± 11.25	36	0.87 ± 0.01
bowtie2	fluxcapacitor	72	35	30.24 ± 6.95	37	0.85 ± 0.04
bowtie2	cufflinks1	80	38	32.99 ± 12.91	43	0.81 ± 0.03
bowtie2	cufflinks2	80	39	39.63 ± 19.74	41	0.83 ± 0.03

Table S5: Average rankings of the pipelines across the data sets with single-end reads. The overall rank was obtained by summing the rankings on each metric. The average value and standard deviation accross datasets is also shown for each metric. The table is sorted by overall rank (top corresponds to lowest rank values).

Pipeline		Overall	Error		Spearman	
Aligner	Quant. Method	Rank	Rank	mean ± sd	Rank	mean ± sd
tophat1	htseq-ine	12	9	17.65 ± 2.5	3	0.94 ± 0.00
gsnap	htseq-ine	15	8	17.59 ± 2.61	7	0.94 ± 0.00
osa	htseq-ine	15	9	17.68 ± 2.55	6	0.94 ± 0.00
tophat2	htseq-ine	19	11	19.31 ± 5.07	8	0.94 ± 0.00
star	htseq-ine	21	11	17.99 ± 2.8	10	0.94 ± 0.00
osa	fluxcapacitor	23	21	20.51 ± 2.67	2	0.95 ± 0.00
tophat1	fluxcapacitor	23	20	20.04 ± 2.82	3	0.95 ± 0.00
osa	cufflinks2	27	12	21.14 ± 9.91	15	0.93 ± 0.01
smalt	htseq-ine	27	12	19.55 ± 6.19	14	0.93 ± 0.00
tophat1	cufflinks2	29	15	23.25 ± 13.86	14	0.93 ± 0.01
star	fluxcapacitor	31	24	21.9 ± 3.22	7	0.94 ± 0.00
gsnap	cufflinks2	32	14	24.72 ± 18.34	18	0.92 ± 0.04
osa	cufflinks1	33	11	23.18 ± 15.1	22	0.92 ± 0.01
star	cufflinks2	33	15	18.15 ± 3.69	18	0.93 ± 0.01
bwa1	htseq-ine	34	19	23.05 ± 7.14	14	0.92 ± 0.02
star	cufflinks1	34	10	17.98 ± 4.16	24	0.92 ± 0.01
tophat1	cufflinks1	34	16	26.65 ± 19.29	18	0.92 ± 0.01
tophat1	htseq-u	34	17	21.06 ± 4.81	17	0.93 ± 0.00
tophat2	cufflinks2	35	13	20.65 ± 7.89	22	0.92 ± 0.01
gsnap	fluxcapacitor	36	24	23.56 ± 3.21	12	0.93 ± 0.00
osa	htseq-u	36	16	21.13 ± 8.71	20	0.92 ± 0.00
tophat2	fluxcapacitor	36	24	24.36 ± 5.37	11	0.93 ± 0.01
gsnap	cufflinks1	38	14	26.06 ± 18.71	24	0.9 ± 0.05
smalt	htseq-u	41	16	19.46 ± 3.48	25	0.92 ± 0.00
bwa2	cufflinks2	42	23	29.01 ± 19.91	19	0.91 ± 0.07
gsnap	htseq-u	42	18	26.48 ± 16.79	24	0.92 ± 0.01
tophat2	htseq-u	42	19	22.34 ± 6.28	22	0.92 ± 0.00
star	htseq-u	45	20	20.18 ± 5.97	25	0.92 ± 0.00
tophat2	cufflinks1	45	16	27.62 ± 23.49	29	0.89 ± 0.09
bwa1	htseq-u	47	27	26.72 ± 7.57	20	0.92 ± 0.02
bwa2	cufflinks1	47	24	34.07 ± 22.23	23	0.89 ± 0.08
smalt	cufflinks2	48	21	21.87 ± 7.75	27	0.91 ± 0.02
smalt	cufflinks1	50	21	23.94 ± 10.8	29	0.91 ± 0.02
bwa1	cufflinks2	51	27	29.56 ± 12.05	25	0.89 ± 0.05
bwa1	cufflinks1	52	26	34.92 ± 17.92	26	0.88 ± 0.05
bwa1	fluxcapacitor	58	32	35.37 ± 11.82	26	0.88 ± 0.05
smalt	fluxcapacitor	58	28	30.21 ± 8.77	30	0.89 ± 0.03
bowtie2	htseq-ine	67	32	37.74 ± 11.35	35	0.84 ± 0.04
bowtie1	fluxcapacitor	69	35	39.95 ± 11.73	34	0.85 ± 0.03
bowtie2	htseq-u	71	34	41.58 ± 14.96	37	0.83 ± 0.04
bowtie2	cufflinks2	73	35	46.67 ± 22.7	38	0.8 ± 0.09
bowtie2	fluxcapacitor	73	35	39.02 ± 7.66	38	0.81 ± 0.05
bowtie2	cufflinks1	74	35	45.36 ± 20.72	39	0.8 ± 0.06

Table S6: Average rankings of the pipelines across the data sets with paired-end reads. The overall rank was obtained by summing the rankings on each metric. The average value and standard deviation accross datasets is shown for each metric between brackets. The table is sorted by overall rank (top corresponds to lowest rank values).

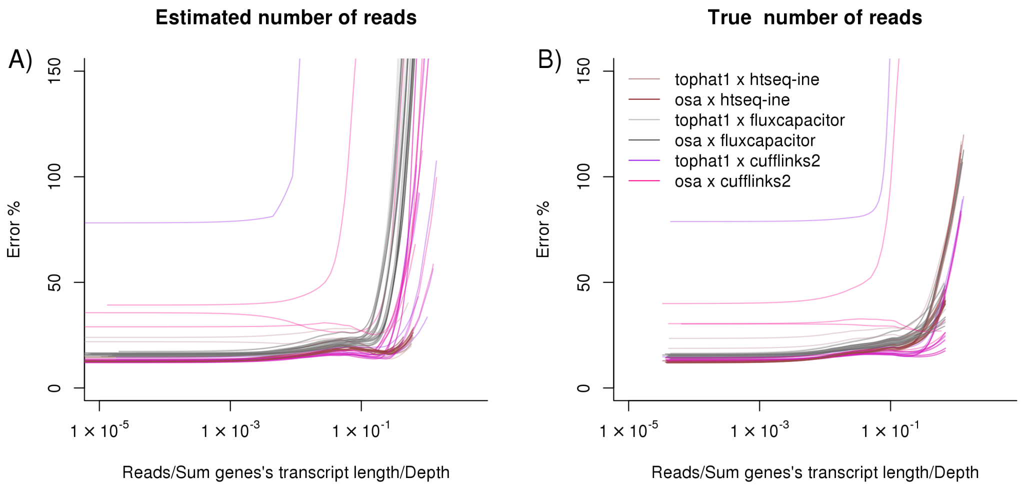

Figure S7: Error by number of reads (normalized per gene using the sum of the transcript lengths of a gene and and sequencing depth of the data set) for multiple pipelines and 16 data sets (single-end). The lines shown are lowess regressions of the errors per gene and data set. A - number of reads per gene used was inferred by the pipeline; B - the number of reads used corresponds to the true number of reads per gene.

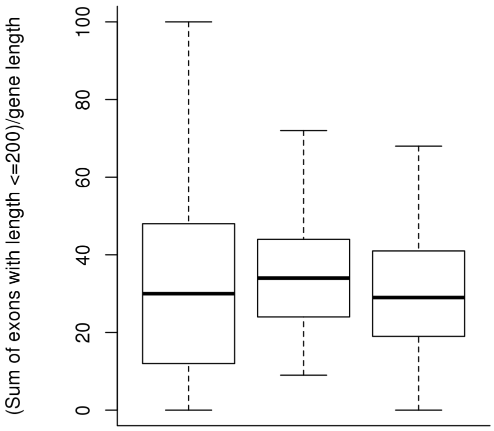

Figure S8: Percentage of the gene length “explained” by exons with a length shorter than 200 nucleotides.

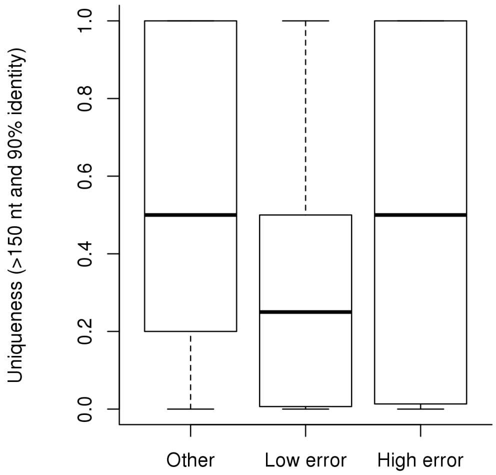

Figure S9: Gene sequence uniqueness: $\frac{1}{N}$, where N is the number of locations in the genome similar to the gene's sequence.

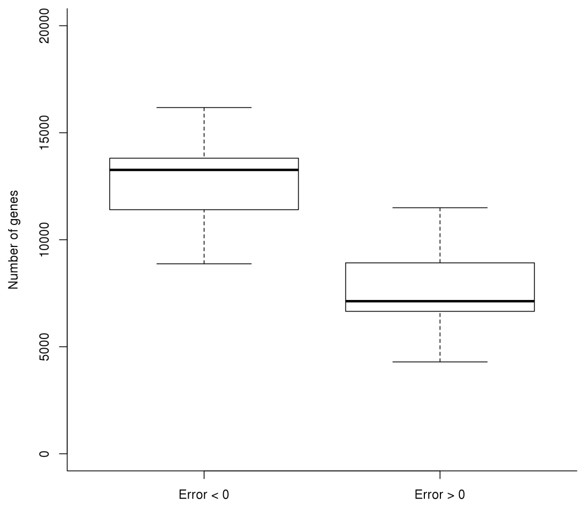

Figure S10: Number of genes with a positive or negative error across all data sets and pipelines.

Ensembl ID	Name	GC %	Chr	N. Trans.	Length
ENSG00000166295	ANAPC16 - anaphase promoting complex subunit 16	43.40	10	1	2814
ENSG00000089006	SNX5 - sorting nexin 5	42.13	20	11	4973
ENSG00000101294	HM13 - histocompatibility (minor) 13	49.35	20	5	5928
ENSG00000171863	RPS7 - ribosomal protein S7	44.76	2	4	5581
ENSG00000163541	SUCLG1 - succinate-CoA ligase, alpha subunit	39.25	2	5	4273
ENSG00000177082	WDR73 - WD repeat domain 73	49.15	15	4	6135
ENSG00000140553	UNC45A - unc-45 homolog A (C. elegans)	53.82	15	4	7930
ENSG00000082068	WDR70 - WD repeat domain 70	39	5	2	7021
ENSG00000197375	SLC22A5 - solute carrier family 22 (organic cation/carnitine transporter), member 5	48.32	5	9	12778
ENSG00000011485	PPP5C - protein phosphatase 5, catalytic subunit	50.75	19	20	7015
ENSG00000213930	GALT - galactose-1-phosphate uridylyltransferase	50.16	9	9	4359
ENSG00000213213	KIAA1984 - KIAA1984	59.33	9	2	6065
ENSG00000197070	ARRDC1 - arrestin domain containing 1	60.84	9	8	3816
ENSG00000168676	KCTD19 - potassium channel tetramerisation domain containing 19	47.44	16	5	7366
ENSG00000103187	COTL1 - coactosin-like 1 (Dictyostelium)	50.24	16	2	8839
ENSG00000122566	HNRNPA2B1 - heterogeneous nuclear ribonucleoprotein A2/B1	40.07	7	6	7532
ENSG00000106258	CYP3A5 - cytochrome P450, family 3, subfamily A, polypeptide 5	40.47	7	5	6717
ENSG00000105971	CAV2 - caveolin 2	37.86	7	3	6633
ENSG00000154438	ASZ1 - ankyrin repeat, SAM and basic leucine zipper domain containing 1	35.27	7	11	3052
ENSG00000196329	GIMAP5 - GTPase, IMAP family member 5	43.79	7	1	6026
ENSG00000198912	C1orf174 - chromosome 1 open reading frame 174	49.32	1	1	4384
ENSG00000142920	ADC - arginine decarboxylase	45.93	1	20	6550
ENSG00000116898	MRPS15 - mitochondrial ribosomal protein S15	49.28	1	1	2908
ENSG00000159214	CCDC24 - coiled-coil domain containing 24	57.95	1	1	3548
ENSG00000126088	UROD - uroporphyrinogen decarboxylase	52.29	1	3	2905
ENSG00000117481	NSUN4 - NOP2/Sun domain family, member 4	47.35	1	4	8461
ENSG00000187889	C1orf168 - chromosome 1 open reading frame 168	38.48	1	1	4611
ENSG00000203965	EFCAB7 - EF-hand calcium binding domain 7	35.15	1	1	5688
ENSG00000125462	C1orf61 - chromosome 1 open reading frame 61	51.53	1	5	8378
ENSG00000127074	RGS13 - regulator of G-protein signaling 13	35.75	1	2	5864
ENSG00000159176	CSRP1 - cysteine and glycine-rich protein 1	50.68	1	7	10311
ENSG00000134548	C12orf39 - chromosome 12 open reading frame 39	38.50	12	2	2925
ENSG00000111786	SRSF9 - serine/arginine-rich splicing factor 9	46.53	12	3	3740
ENSG00000204348	DOM3Z - dom-3 homolog Z (C. elegans)	58.72	6	5	2482
ENSG00000114857	NKTR - natural killer-tumor recognition sequence	37.54	3	4	17336
ENSG00000237765	FAM200B - family with sequence similarity 200, member B	40.86	4	3	4812
ENSG00000157379	DHRS1 - dehydrogenase/reductase (SDR family) member 1	48.04	14	6	4994
ENSG00000054690	PLEKHH1 - pleckstrin homology domain containing, family H (with MyTH4 domain) member 1	48.02	14	14	10788
ENSG00000185189	NRBP2 - nuclear receptor binding protein 2	62.45	8	6	4921
ENSG00000133812	SBF2 - SET binding factor 2	38.31	11	18	16722
ENSG00000109920	FNBP4 - formin binding protein 4	43.43	11	2	7581
ENSG00000187066	AP003068.6.1	55.56	11	3	4227
ENSG00000149294	NCAM1 - neural cell adhesion molecule 1	41.71	11	41	12734

Table S7: Genes with consistent high error (greater than 100%) across most pipelines and data sets: Ensembl gene ID; Gene name; percentage of GC-content; location (Chromossome); number of transcripts; gene length (sum of the length of the exons).

Ensembl ID	GO term
ENSG00000166295	protein ubiquitination;protein ubiquitination;mitosis;cell division;cytoplasm;anaphase-promoting complex
ENSG00000089006	pinocytosis;cell communication;protein transport;ruffle;phagocytic cup;cytoplasmic vesicle membrane;extrinsic to internal side of plasma membrane;extrinsic to endosome membrane;early endosome membrane;macropinocytic cup;phosphatidylinositol binding;phosphatidylinositol binding
ENSG00000101294	membrane protein proteolysis;plasma membrane;endoplasmic reticulum;rough endoplasmic reticulum;cell surface;integral to cytosolic side of endoplasmic reticulum membrane;integral to lumenal side of endoplasmic reticulum membrane;protein binding;peptidase activity;aspartic endopeptidase activity, intramembrane cleaving;protein homodimerization activity
ENSG00000171863	nuclear-transcribed mRNA catabolic process, nonsense-mediated decay;rRNA processing;translation;translation;translation;translational initiation;translational elongation;translational termination;SRP-dependent cotranslational protein targeting to membrane;viral reproduction;gene expression;RNA metabolic process;mRNA metabolic process;viral infectious cycle;viral transcription;ribosomal small subunit biogenesis;cellular protein metabolic process;cytosolic small ribosomal subunit;cytosolic small ribosomal subunit;ribonucleoprotein complex;cytosol;ribosome;nucleus;nucleolus;microtubule organizing center;90S preribosome;small-subunit processome;protein binding;RNA binding;structural constituent of ribosome
ENSG00000163541	tricarboxylic acid cycle;tricarboxylic acid cycle;succinyl-CoA metabolic process;succinate metabolic process;small molecule metabolic process;plasma membrane;mitochondrion;cytoplasm;mitochondrial inner membrane;mitochondrial matrix;succinate-CoA ligase complex (GDP-forming);ATP citrate synthase activity;succinate-CoA ligase (ADP-forming) activity;succinate-CoA ligase (GDP-forming) activity;GTP binding;GDP binding;protein heterodimerization activity;cofactor binding
ENSG00000140553	muscle organ development;cell differentiation;chaperone-mediated protein folding;nucleus;perinuclear region of cytoplasm;Hsp90 protein binding
ENSG00000197375	sodium ion transport;drug transmembrane transport;quaternary ammonium group transport;carnitine transport;carnitine transport;drug transport;quorum sensing involved in interaction with host;transmembrane transport;positive regulation of intestinal epithelial structure maintenance;sodium-dependent organic cation transport;plasma membrane;plasma membrane;integral to membrane;basolateral plasma membrane;apical plasma membrane;brush border membrane;brush border membrane;protein binding;ATP binding;carnitine transporter activity;carnitine transporter activity;drug transmembrane transporter activity;symporter activity;quaternary ammonium group transmembrane transporter activity;PDZ domain binding;antibiotic transporter activity
ENSG00000011485	signal transduction;transcription, DNA-dependent;protein dephosphorylation;mitosis;positive regulation of I-kappaB kinase/NF-kappaB cascade;response to morphine;cytosol;nucleus;cytoplasm;Golgi apparatus;neuron projection;neuronal cell body;protein binding;protein serine/threonine phosphatase activity;signal transducer activity;metal ion binding;identical protein binding
ENSG00000213930	carbohydrate metabolic process;galactose metabolic process;UDP-glucose catabolic process;galactose catabolic process;small molecule metabolic process;cytosol;Golgi apparatus;UDP-glucose:hexose-1-phosphate uridylyltransferase activity;zinc ion binding
ENSG00000168676	protein homooligomerization
ENSG00000103187	defense response to fungus;biological_process;cellular_component;cytoplasm;cytoskeleton;protein binding;actin binding;enzyme binding
ENSG00000122566	nuclear mRNA splicing, via spliceosome;nuclear mRNA splicing, via spliceosome;mRNA processing;RNA splicing;gene expression;RNA transport;ribonucleoprotein complex;nucleus;cytoplasm;nucleoplasm;spliceosomal complex;nucleolus;heterogeneous nuclear ribonucleoprotein complex;catalytic step 2 spliceosome;nucleotide binding;protein binding;RNA binding;single-stranded telomeric DNA binding
ENSG00000106258	xenobiotic metabolic process;steroid metabolic process;alkaloid catabolic process;drug catabolic process;small molecule metabolic process;oxidative demethylation;endoplasmic reticulum membrane;electron carrier activity;monooxygenase activity;oxidoreductase activity;oxygen binding;heme binding;aromatase activity
ENSG00000105971	negative regulation of endothelial cell proliferation;vesicle fusion;mitochondrion organization;endoplasmic reticulum organization;regulation of mitosis;synaptic transmission;vesicle organization;positive regulation of dopamine receptor signaling pathway;vesicle docking;skeletal muscle fiber development;protein oligomerization;caveola assembly;plasma membrane;Golgi membrane;intracellular;acrosomal membrane;cytosol;integral to plasma membrane;nucleus;Golgi apparatus;transport vesicle;lipid particle;caveola;cell surface;extrinsic to internal side of plasma membrane;protein complex;membrane raft;perinuclear region of cytoplasm;protein binding;syntaxin binding;D1 dopamine receptor binding;protein homodimerization activity;phosphoprotein binding
ENSG00000154438	signal transduction;male meiosis;multicellular organismal development;spermatogenesis;cell differentiation;gene silencing by RNA;piRNA metabolic process;DNA methylation involved in gamete generation;cytoplasm;pi-body;signal transducer activity

Table S8: GO terms of the genes with consistent high error (greater than 100%) across most pipelines and data sets (part 1/2).

Ensembl ID	GO term
ENSG00000196329	temperature homeostasis;positive regulation of natural killer cell cytokine production;positive regulation of humoral immune response mediated by circulating immunoglobulin;positive regulation of calcium ion transport into cytosol;T cell differentiation;negative regulation of interferon-gamma production;positive regulation of CD4-positive, CD25-positive, alpha-beta regulatory T cell differentiation;myeloid dendritic cell differentiation;T cell homeostasis;negative regulation of apoptotic process;negative regulation of nitric oxide biosynthetic process;positive regulation of gamma-delta T cell differentiation;positive regulation of membrane potential;positive regulation of natural killer cell mediated cytotoxicity;regulation of mitochondrial membrane permeability;negative regulation of T cell activation;negative regulation of lipid catabolic process;integral to membrane;lysosome;mitochondrial outer membrane;GTP binding
ENSG00000198912	nucleus
ENSG00000142920	ornithine metabolic process;polyamine metabolic process;polyamine biosynthetic process;spermatogenesis;cellular nitrogen compound metabolic process;small molecule metabolic process;agmatine biosynthetic process;mitochondrion;cytosol;arginine decarboxylase activity
ENSG00000116898	translation;mitochondrion;mitochondrial small ribosomal subunit;nuclear membrane;structural constituent of ribosome
ENSG00000126088	liver development;porphyrin-containing compound metabolic process;protoporphyrinogen IX biosynthetic process;heme biosynthetic process;heme biosynthetic process;response to iron ion;response to organic cyclic compound;response to amine stimulus;response to mercury ion;response to estradiol stimulus;small molecule metabolic process;response to ethanol;uroporphyrinogen III metabolic process;response to methylmercury;response to fungicide;cellular response to arsenic-containing substance;cytosol;nucleus;cytoplasm;microtubule cytoskeleton;uroporphyrinogen decarboxylase activity;uroporphyrinogen decarboxylase activity;ferrous iron binding
ENSG00000117481	mitochondrial large ribosomal subunit;methyltransferase activity
ENSG00000203965	calcium ion binding
ENSG00000125462	nucleus
ENSG00000127074	G-protein coupled receptor signaling pathway;termination of G-protein coupled receptor signaling pathway;positive regulation of GTPase activity;plasma membrane;cytosol;nucleus;cytoplasm;GTPase activator activity
ENSG00000159176	nucleus;zinc ion binding
ENSG00000134548	extracellular region;nucleus;intracellular membrane-bounded organelle;transport vesicle
ENSG00000111786	nuclear mRNA splicing, via spliceosome;transcription from RNA polymerase II promoter;termination of RNA polymerase II transcription;mRNA splice site selection;mRNA processing;mRNA export from nucleus;RNA splicing;gene expression;mRNA 3-end processing;negative regulation of nuclear mRNA splicing, via spliceosome;nucleoplasm;nucleotide binding;RNA binding
ENSG00000204348	nucleotide binding;metal ion binding
ENSG00000114857	protein folding;membrane;peptidyl-prolyl cis-trans isomerase activity;cyclosporin A binding
ENSG00000237765	nucleic acid binding
ENSG00000157379	endoplasmic reticulum;mitochondrial inner membrane;nucleotide binding;oxidoreductase activity
ENSG00000054690	cytoskeleton;phospholipid binding
ENSG00000185189	negative regulation of macroautophagy;neuron differentiation;negative regulation of neuron apoptotic process;cytoplasm
ENSG00000133812	myelination;protein tetramerization;membrane;vacuolar membrane;protein binding;phosphatase activity;phosphatase regulator activity;phosphatase binding;phosphatidylinositol binding;protein homodimerization activity
ENSG00000149294	cell adhesion;axon guidance;cytokine-mediated signaling pathway;homotypic cell-cell adhesion;positive regulation of calcium-mediated signaling;interferon-gamma-mediated signaling pathway;plasma membrane;Golgi membrane;integral to membrane;extracellular region;external side of plasma membrane;cell surface;anchored to membrane;axon;neuronal cell body

Table S9: GO terms for the genes with consistent high error (greater than 100%) across most pipelines and data sets (part 2/2).
